# High-throughput electron tomography identifies centriole over-elongation as an early event in plasma cell disorders

**DOI:** 10.1038/s41375-023-02056-y

**Published:** 2023-10-11

**Authors:** Sebastian Köhrer, Tobias Dittrich, Martin Schorb, Niels Weinhold, Isabella Haberbosch, Mandy Börmel, Gabor Pajor, Hartmut Goldschmidt, Carsten Müller-Tidow, Marc S. Raab, Lukas John, Anja Seckinger, Alexander Brobeil, Peter Dreger, Tamás Tornóczky, László Pajor, Ute Hegenbart, Stefan O. Schönland, Yannick Schwab, Alwin Krämer

**Affiliations:** 1 Clinical Cooperation Unit Molecular Hematology/Oncology, German Cancer Research Center (DKFZ) and Department of Internal Medicine V, University of Heidelberg, Heidelberg, Germany; 2 Cell Biology and Biophysics Unit, European Molecular Biology Laboratory (EMBL), Heidelberg, Germany; 3 Department of Internal Medicine V, University of Heidelberg, Heidelberg, Germany; 4 Amyloidosis Center, University of Heidelberg, Heidelberg, Germany; 5 Electron Microscopy Core Facility, European Molecular Biology Laboratory (EMBL), Heidelberg, Germany; 6 Department of Pathology, University of Pécs Medical School and Clinic, Pécs, Hungary; 7 Department of Internal Medicine V, GMMG-Studygroup at University of Heidelberg, Heidelberg, Germany; 8 National Center for Tumor Diseases (NCT), University of Heidelberg, Heidelberg, Germany; 9 Institute of Pathology, University of Heidelberg, Heidelberg, Germany

**Keywords:** Myeloma, Cell biology

## Abstract

Plasma cell disorders are clonal outgrowths of pre-malignant or malignant plasma cells, characterized by extensive chromosomal aberrations. Centrosome abnormalities are a major driver of chromosomal instability in cancer but their origin, incidence, and composition in primary tumor cells is poorly understood. Using cutting-edge, semi-automated high-throughput electron tomography, we characterized at nanoscale 1386 centrioles in CD138^pos^ plasma cells from eight healthy donors and 21 patients with plasma cell disorders, and 722 centrioles from different control populations. In plasma cells from healthy individuals, over-elongated centrioles accumulated with age. In plasma cell disorders, centriole over-elongation was notably frequent in early, pre-malignant disease stages, became less pronounced in overt multiple myeloma, and almost entirely disappeared in aggressive plasma cell leukemia. Centrioles in other types of patient-derived B cell neoplasms showed no over-elongation. In contrast to current belief, centriole length appears to be highly variable in long-lived, healthy plasma cells, and over-elongation and structural aberrations are common in this cell type. Our data suggest that structural centrosome aberrations accumulate with age in healthy CD138^pos^ plasma cells and may thus play an important role in early aneuploidization as an oncogenic driver in plasma cell disorders.

## Introduction

Chromosomal instability (CIN) is a hallmark of cancer [1]. It increases the probability of oncogenic events and creates a heterogeneous cell population with enhanced abilities to adapt and evolve. CIN results from errors in mitotic chromosome separation, with centrosome aberrations (CA) being the most prominent cause of mis-segregation [2–4].

The centrosome is the microtubule-organizing center in most animal cells. It plays a major role in various cellular actions including ciliogenesis and cell division. During mitosis, centrosomes ensure accurate chromosome segregation by forming the bipolar spindle apparatus [5]. Centrosomes consist of a pair of microtubule-based cylinders, the centrioles, which are embedded in a protein matrix called the pericentriolar material [2, 3, 6]. Centrioles have a physiological length of up to 500 nm, a diameter of approximately 200 nm, and show a typical 9 × 3 microtubule-triplet architecture, which is highly conserved among different species [7–10]. To enable proper spindle formation, centrioles duplicate in S phase starting with the formation of a new daughter centriole next to each preexisting mother centriole. These daughter centrioles subsequently mature and elongate until mitosis. Mother centrioles, as opposed to daughter centrioles, are decorated with two types of appendages, one set of nine distal appendages and a variable amount of subdistal appendages, with the latter being possibly located along the entire length of the centriole [10, 11]. Correct centriole number and structure are essential for accurate chromosome segregation and are thus tightly controlled in proliferating, non-transformed cells by different classes of proteins such as scaffold proteins and centriole elongation activators and inhibitors [5–7, 12].

Abnormalities in centrosome structure and number have been reported in various solid tumors and hematological malignancies and are associated with CIN and poor prognosis [2, 4, 13–16]. CA are believed to represent an early event in the evolution of malignant phenotypes in organotypic culture and animal models. They have been detected in early disease stages such as in situ carcinomas and can occur before TP53 loss [4, 15]. However, clear evidence on how CA develop is lacking.

Plasma cell disorders (PCD) include pre-malignant monoclonal gammopathies of undetermined significance (MGUS) and develop via asymptomatic smoldering myeloma (SMM) to overt multiple myeloma (MM) and highly aggressive plasma cell leukemia (PCL) [17, 18]. Whilst disease-defining mutations are lacking, chromosome aberrations such as immunoglobulin heavy chain translocations and hyperdiploidy are considered initiating events and are prevalent from earliest disease stages onwards [17, 18]. It is estimated that these events occur approximately 20–40 years prior to diagnosis, at an age between 20–30 years [19].

Most studies on CA in primary tumor tissues rely on single antigen immunostainings against pericentriolar matrix proteins at low resolution. Virtually nothing is known on the detailed composition of CA in primary tumor cells at electron microscopy (EM) resolution. We have therefore recently developed a high-throughput electron tomography (ET) workflow that allows for the visualization of the centriole structure in patient-derived cells at nanoscale and described plasma cells with over-elongated centrioles in one case of relapsed/refractory multiple myeloma by applying this technology [20]. To further enhance our understanding on the origin and evolution of CA in primary cancer cells, we now chose the spectrum of PCD as a paradigm for malignant progression from a precursor lesion to aggressive malignancy for analysis by cutting-edge semi-automated high-throughput ET [20].

## Materials and methods

### Cell lines

U2OS cells (HTB-96) were obtained from ATCC (Manassas, Virginia, USA). Polo-like kinase 4 (PLK4) overexpression was achieved in U2OS cells as described previously [21]

### Patient samples

Bone marrow aspirates of 21 patients with PCD and 8 healthy donors were acquired according to clinical standard operating procedures at the Department of Internal Medicine V, University of Heidelberg. Furthermore, peripheral blood from 1 patient with B-cell acute lymphoblastic leukemia (B-ALL) and 3 patients with B-cell chronic lymphocytic leukemia (B-CLL) was collected. Five additional bone marrow samples from patients with B-ALL were obtained according to clinical standard operating procedures at the University of Pécs Medical Center. All donors gave written informed consent, and the study was performed in accordance with the principles of the Declaration of Helsinki. The Ethics Committee of the University of Heidelberg, the European Molecular Biology Laboratory (EMBL) review board, and the Hungarian Research Ethics Committee of the Medical Research Council all approved the study (Ethics Committee of the University of Heidelberg approval reference number: S-206/2011; EMBL BIAC application number: 2019-005; Hungarian Research Ethics Committee of the Medical Research Council reference number: BMEÜ/4146/2022/EKU). Detailed de-identified patient information can be found in Table 1 and Supplementary Table S1.

**Table 1:** Baseline characteristics of patients with plasma cell disorders.

Description	MGUS	SMM	MM	PCL
*N* patients	4	3	12	2
Gender = female	1 (25.0)	0 (0.0)	1 (8.3)	1 (50.0)
Age, years	69.0 [62.0, 81.0]	57.0 [57.0, 64.0]	67.0 [57.0, 76.0]	63.5 [58.0, 69.0]
Prior lines of therapy	0.0 [0.0, 0.0]	0.0 [0.0, 0.0]	4.5 [0.0, 8.0]	6.5 [6.0, 7.0]
HDM + ASCT	0 (0.0)	0 (0.0)	10 (83.3)	2 (100.0)
BM plasmocytosis, %	8.4 [7.1, 9.1]	18.7 [11.5, 21.3]	58.7 [27.5, 91.0]	73.2 [50.0, 96.4]
Ki67+ PC, %	22.1 [16.0, 40.5]	27.3 [24.1, 39.4]	37.3 [6.4, 93.3]	78.9^a^
Salmon–Durie stage, I-II-III			0 (0.0) - 1 (8.3) - 10 (82.3)	
Type of HC				
A	0 (0.0)	0 (0.0)	1 (8.3)	0 (0.0)
G	2 (50.0)	2 (66.7)	8 (66.7)	1 (50.0)
None	2 (50.0)	1 (33.3)	3 (25.0)	1 (50.0)
Type of LC = lambda	2 (50.0)	1 (33.3)	3 (25.0)	1 (50.0)
Monoclonal component, g/l	3.1 [3.1, 3.1]	6.0 [2.1, 10.0]	16.5 [3.8, 39.9]	4.2 [4.2, 4.2]

Categorical data is shown as count (% of respective total), continuous data is shown as median [range]. BM plasmocytosis, fraction of plasma cells in bone marrow leukocytes.

*HC* heavy chain measured in serum, *LC* light chain measured in serum, *HDM* *+* *ASCT* high-dose chemotherapy with melphalan followed by autologous stem cell transplantation.

^a^This parameter was only available for one patient with PCL.

### Immunofluorescence imaging

CD138^pos^ plasma cells from patients with PCD and healthy control donors, CD138^neg^ bone marrow mononuclear cells from 3 healthy control donors, B-ALL and B-CLL patient samples, and U2OS-PLK4 cells were prepared for immunofluorescence microscopy analysis of numerical centrosome aberrations by staining with centrin (04-1624, Merck Millipore, Burlington, Massachusetts, USA) and pericentrin (ab4448, Abcam, Cambridge, UK) antibodies.

### High-throughput electron microscopy and tomography

High-throughput electron tomography was performed as described recently [20, 22]. Sample preparation for ET included chemical fixation, post-fixation with osmium tetroxide and uranyl acetate, resin embedding, and serial sectioning. A semi-automated high-throughput EM workflow was used to screen for centrosome-containing cells. After identification of centrioles, respective cells were targeted on the remaining sections semi-automatically and single-axis ET was performed at 15,500× magnification (1.55 nm/px). Reconstruction and joining of raw data yielded 3D volumes of approximately 3.1 µm × 3.1 µm in X and Y, and at least 1 µm in Z dimension, respectively. Per case, a minimum of 30 completely featured centrioles was evaluated. Centriole parameters were measured manually using the model feature in IMOD, and statistical evaluation was achieved in R statistical environment 3.5.3.

### Semi-automated MUM1-based quantification of histological sections

In 16 out of 21 patients with PCD, bone marrow histology samples were available. For immunohistochemical assessment of proliferation activity, a representative tissue block containing MUM1-positive myeloma cells was selected. After immunohistochemical staining for MUM1 and Ki-67, images were acquired and evaluated using NDP.view2 software suite (Hamamatsu, Herrsching, Germany).

### Gene expression profiling

Expression data were generated based on available material from the concluded GMMG HD-4 and MM5 trials [23]. RNA extraction was performed using the RNeasy Mini Kit (74104, Qiagen, Venlo, Netherlands), the SV Total RNA Isolation System (Z3101, Promega, Madison, Wisconsin, USA) and Trizol RNA extraction reagent (15596026, Invitrogen, Waltham, Massachusetts, USA). Labeled cRNA was generated using the small sample labeling protocol vII (Affymetrix, Santa Clara, California, USA), and hybridized to GeneChip™ Human Genome U133 Plus 2.0 Arrays (900466, Applied Biosystems, Waltham, Massachusetts, USA), according to the manufacturer’s instructions.

## Results

### High-throughput electron tomography enables establishment of a terabyte-scale database of centrioles in primary human cells

Using our high-throughput ET workflow, we screened a total of 48,672 cells by transmission EM, yielding volumetric EM data of 2108 completely pictured centrioles in 1439 cells, including 1386 centrioles in 988 bone-marrow derived CD138^pos^ cells from 21 patients with plasma cell disorders (PCD) and 8 healthy donors (Table 2, Supplementary Tables S2 and S3). Of the PCD patients, four, three, twelve, and two suffered from MGUS, SMM, MM, and PCL, respectively. Baseline characteristics of patients with PCD are presented in Table 1. Non-plasma cell controls comprised 205 completely pictured centrioles in 136 CD138^neg^ bone marrow mononuclear cells from 3 healthy donors, 380 centrioles in 262 leukemic B-cells from 2 patients with B-CLL and 6 patients with B-ALL, and 137 centrioles in 53 U2OS cells conditionally over-expressing PLK4, the principal kinase regulating centriole duplication [2–4, 10]. The complete database of 2.48 TB can be visualized interactively via MoBIE [24] as described in the supplement.

**Table 2:** Centriole parameters of patients with plasma cell disorders and controls.

Description	CD138-BMNC	U2OS-PLK4	B-ALL	B-CLL	Healthy PC	MGUS	SMM	MM	PCL
*N* biological samples	3	3	6	2	8	4	3	12	3
*N* cells	30.0 [24.0, 82.0]	19.0 [13.0, 21.0]	29.5 [19.0, 63.0]	31.5 [29.0, 34.0]	23.5 [19.0, 32.0]	28.5 [23.0, 37.0]	25.0 [18.0, 28.0]	30.5 [20.0, 179.0]	26.0 [18.0, 39.0]
*N* centrioles	47.0 [33.0, 125.0]	49.0 [18.0, 70.0]	42.0 [31.0, 86.0]	45.5 [39.0, 52.0]	33.5 [30.0, 40.0]	37.5 [34.0, 49.0]	34.0 [30.0, 37.0]	41.5 [32.0, 250.0]	39.0 [24.0, 50.0]
*N* centrioles per centrosome	1.5 [1.4, 1.6]	2.6 [1.4, 3.3]	1.5 [1.4, 1.6]	1.4 [1.3, 1.5]	1.4 [1.2, 1.8]	1.4 [1.2, 1.5]	1.5 [1.2, 1.7]	1.4 [1.2, 1.6]	1.3 [1.3, 1.5]
Mothers (%)	45.5 [42.6, 50.4]	24.5 [22.9, 38.9]	54.2 [44.2, 56.4]	46.5 [44.2, 48.7]	53.2 [44.7, 63.9]	54.0 [47.1, 62.9]	50.0 [48.6, 53.3]	52.1 [32.4, 64.0]	61.5 [52.0, 62.5]
Median centriole length, nm	377.0 [328.9, 377.1]	345.1 [334.2, 349.6]	336.7 [317.9, 361.6]	390.0 [374.7, 405.2]	592.0 [470.5, 713.6]	534.6 [531.3, 650.5]	511.0 [442.5, 1050.5]	415.7 [339.7, 495.9]	347.5 [328.9, 373.3]
Maximal centriole length, nm	511.0 [475.3, 752.6]	521.0 [495.9, 573.0]	477.6 [403.1, 596.0]	572.5 [511.0, 634.0]	1686.9 [1148.1, 2557.2]	1751.1 [1475.3, 2489.3]	1300.8 [1235.4, 2570.5]	967.1 [513.0, 1740.2]	585.8 [442.5, 648.1]
Median centriole diameter, nm	204.1 [203.6, 205.7]	197.4 [191.2, 206.8]	204.7 [199.7, 207.7]	199.7 [198.4, 201.1]	203.3 [199.3, 206.4]	202.8 [202.0, 205.4]	206.7 [203.8, 208.5]	205.7 [202.0, 231.1]	199.4 [199.4, 202.5]
Centrioles > 500 nm, %	2.1 [0.0, 8.0]	4.1 [0.0, 5.6]	0.0 [0.0, 2.6]	11.2 [1.9, 20.5]	75.0 [45.0, 76.3]	61.9 [57.1, 65.7]	51.4 [36.7, 85.3]	23.7 [2.7, 49.0]	4.2 [0.0, 8.0]
Centrioles asymmetric, %	0.0 [0.0, 0.0]	0.0 [0.0, 0.0]	0.0 [0.0, 0.0]	0.0 [0.0, 0.0]	21.2 [8.3, 39.5]	8.1 [5.7, 11.8]	5.4 [0.0, 6.7]	0.0 [0.0, 6.2]	0.0 [0.0, 0.0]
Centrioles broken, %	0.0 [0.0, 0.0]	0.0 [0.0, 0.0]	0.0 [0.0, 0.0]	0.0 [0.0, 0.0]	1.5 [0.0, 10.5]	0.0 [0.0, 2.0]	0.0 [0.0, 0.0]	0.0 [0.0, 3.1]	0.0 [0.0, 0.0]
Centrioles incomplete, %	0.0 [0.0, 0.0]	0.0 [0.0, 0.0]	0.0 [0.0, 0.0]	0.0 [0.0, 0.0]	14.3 [6.7, 26.3]	4.0 [2.0, 17.1]	2.7 [0.0, 11.8]	0.0 [0.0, 8.1]	0.0 [0.0, 0.0]
Centrioles abnormal, %	0.0 [0.0, 0.0]	0.0 [0.0, 0.0]	0.0 [0.0, 0.0]	0.0 [0.0, 0.0]	32.5 [15.6, 55.3]	12.1 [10.2, 20.0]	8.1 [6.7, 11.8]	1.9 [0.0, 9.4]	0.0 [0.0, 0.0]

Data is shown as median [range] of the investigated biological samples. In PCL, for one patient, cells from peripheral blood and bone marrow were analyzed separately. Parameters (e.g., centriole length or maximal centriole length) were first calculated for each individual biological sample. Medians and ranges were then calculated for the respective entities based on these parameters. In case of *n* = 2, the two values given in square brackets represent the medians of parameters calculated separately for the two individuals. Centrioles abnormal, presence of any of the phenotypes (asymmetric, broken, or incomplete). Centriole parameters stratified by mother and daughter centrioles, respectively, are displayed in Supplementary Table S3.

### Plasma cells from healthy donors and patients with plasma cell disorders display normal centriole numbers and diameters

**Fig. 1:**
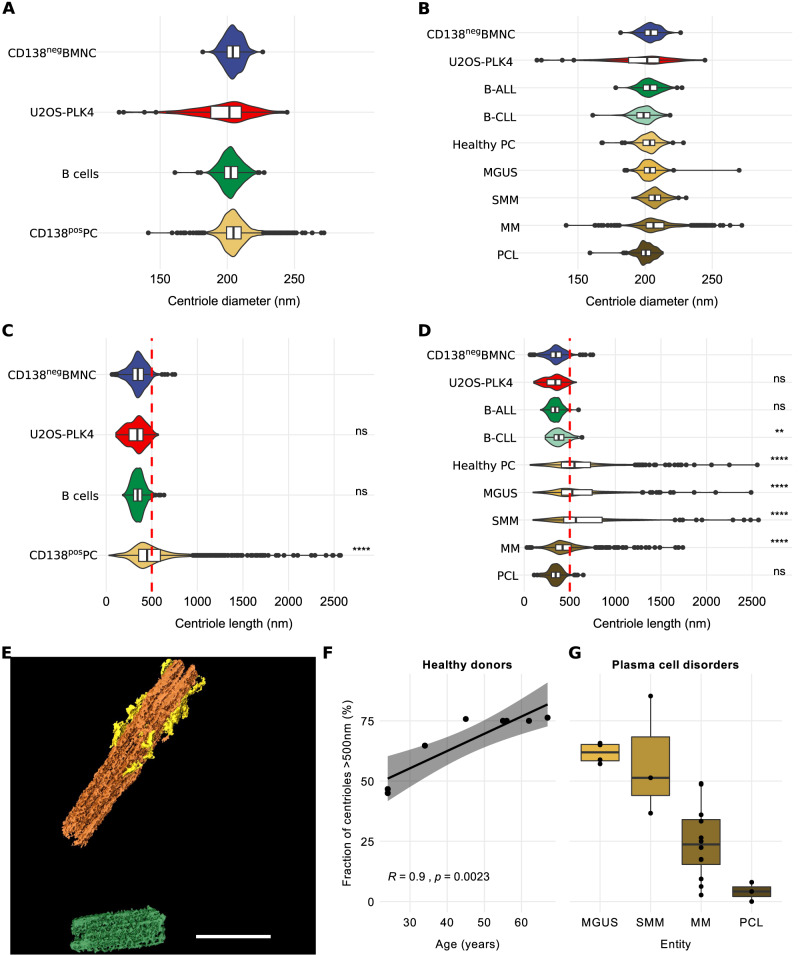
Over-elongation of centrioles increases with age in human plasma cells and decreases with increasingly aggressive stages of plasma cell disorders. **A** Violin plots and integrated box plots showing the distribution of centriole diameters in normal CD138^neg^ bone marrow mononuclear cells, U2OS-PLK4 cells, B-cells from patients with B-ALL or B-CLL, and CD138^pos^ plasma cells from healthy donors and patients with plasma cell disorders. **B** Distribution of centriole diameters in normal CD138^neg^ bone marrow mononuclear cells, U2OS-PLK4, B-cells from patients with B-ALL or B-CLL, and CD138^pos^ plasma cells. Centriole diameters in cells from healthy donors, and patients with MGUS, SMM, MM, and PCL are depicted separately. **C** Violin plots and integrated box plots showing the distribution of centriole lengths of individual centrioles from normal CD138^neg^ bone marrow mononuclear cells, U2OS-PLK4, B-cells from patients with B-ALL or B-CLL, and CD138^pos^ plasma cells from healthy donors and patients with plasma cell disorders. The cut-off value for over-elongation of 500 nm is displayed as red dashed line. **D** Distribution of centriole lengths in normal CD138^neg^ bone marrow mononuclear cells, U2OS-PLK4, B-cells from patients with B-ALL or B-CLL, and CD138^pos^ plasma cells from healthy donors. Centriole lengths in cells from patients with MGUS, SMM, MM, and PCL are depicted separately. The cut-off value for over-elongation of 500 nm is displayed as red dashed line. **E** 3D visualization of an exemplary over-elongated centrosome using Amira-Avizo software. The mother centriole (orange) shows over-elongation (total length: 1654 nm) and supernumerary subdistal appendages (yellow). One over-elongated daughter centriole (length: 712 nm) is displayed in green. Scale Bar, 500 nm. **F** Scatter plot of donor age and fraction of over-elongated centrioles in CD138^pos^ plasma cells from 8 healthy donors. The fitted line, *R* and *p* values were obtained by linear regression. **G** Fraction of over-elongated centrioles in CD138^pos^ plasma cells from MGUS, SMM, MM, and PCL patients displayed as a box plot. For **A**–**D**, individual centriole measures of all biological samples are pooled into one group and statistical analysis were applied to the entire groups. The Wilcoxon rank sum test was used in panels (**C**, **D**) for pairwise comparison of all groups to the CD138^neg^ BMNC, which served as reference. ns not significant. **p < 0.01. ****p < 0.0001. *N* (individual centrioles) for panels **A**–**D**: CD138^neg^ bone marrow mononuclear cells (205), U2OS-PLK4 (137), B-cells (380), CD138^pos^ plasma cells (1386), B-ALL (289), B-CLL (91), healthy plasma cells (275), MGUS (158), SMM (101), MM (739), PCL (113). *N* (biological replicates) for **F** and **G**: healthy donors (8), MGUS (4), MM (12), SMM (3), PCL (3).

Depending on the cell cycle, a centrosome is comprised of either one or two centrioles with a diameter of about 200 nm each [4, 10]. Centrosomal regions acquired by ET in plasma cells from healthy donors and patients with PCD contained a median of 1.42 (range, 1–3) and 1.41 (range, 1–4) centrioles per centrosome, respectively. In contrast, U2OS-PLK4 cells displayed a median of 2.58 (range, 1–8) centrioles per centrosome (Table 2). Concordantly, immunofluorescence microscopy using antibodies against centrin and pericentrin to label centrioles and pericentriolar material revealed supernumerary centrioles in only 2.0% and 3.9% of plasma cells from healthy donors and patients with PCD, respectively, which lies within the range found in healthy cells of B-lymphatic origin [16]. On the contrary, supernumerary centrioles occurred frequently (55.7%) in U2OS cells overexpressing PLK4 as evaluated by immunofluorescence microscopy. Median centriole diameters extracted from ET data were normal in plasma cells from both healthy donors (203 nm, range 199–206 nm) and PCD patients (204 nm, range 199–231 nm) as well as in all control samples (204 nm, range 191–208 nm) examined (Fig. 1A, B, Table 2, Supplementary Tables S2 and S3).

### Centrioles over-elongate with donor age in bone marrow-derived plasma cells from healthy donors

Centriole ultrastructure is believed to be highly conserved across species, with tightly controlled centriole lengths not exceeding 500 nm in human cells [6, 7]. Accordingly, ET revealed a median length of 377 nm (range, 329–377 nm) in 205 centrioles of CD138^neg^ bone marrow mononuclear cells from 3 healthy donors (Fig. 1C, D, Supplementary Table 2, Supplementary Tables S2 and S3). B-CLL, B-ALL, and U2OS-PLK4 cells showed similar median centriole lengths of 390 nm (range, 375–405 nm), 337 nm (range, 318–362 nm), and 345 nm (range, 334–350 nm), respectively. In contrast, CD138^pos^ plasma cells from 8 healthy donors displayed a median centriole length of 592 nm (range, 471–714 nm), with 75% (range, 45–76%) of centrioles being over-elongated, i.e., exceeding a threshold length of 500 nm (Table 2, Supplementary Tables S2 and S3). A segmented example of an over-elongated centriole is shown in Fig. 1E. Interestingly, the fraction of over-elongated centrioles increased with age in healthy donor plasma cells, ranging from 45.0% in the youngest (24 years of age) to 76.3% in the oldest (67 years of age) donor (Fig. 1F).

### Centriole over-elongation is less pronounced in advanced plasma cell disorders

Similar to the rates in plasma cells from healthy donors, 62% (range, 57–66%) and 51% (range, 37–85%) of CD138^pos^ plasma cells from 4 MGUS and 3 SMM patients showed centriole over-elongation, respectively (Fig. 1C, D, G, Table 2, Supplementary Table S2). Median centriole lengths were 535 nm (range, 531–651 nm) in MGUS and 511 nm (range, 443–1051 nm) in SMM patients (Table 2). The longest centrioles in cells from patients with MGUS and SMM measured 2489 nm and 2570 nm, respectively. In patients with overt MM, centriole over-elongation was less pronounced, and almost completely disappeared in PCL (Fig. 1D, G). Only 24% (range, 3–49%) and 4% (range, 0–8%) of centrioles in CD138^pos^ plasma cells from 12 MM and 3 PCL samples were over-elongated, respectively. Median centriole lengths were 416 nm (range, 340–496 nm) in MM and 348 nm (range, 329–373 nm) in PCL (Table 2). The longest centrioles in MM and PCL measured 1740 nm and 648 nm, respectively.

### Proliferative activity of plasma cells correlates inversely with centriole length in plasma cell disorders

**Fig. 2:**
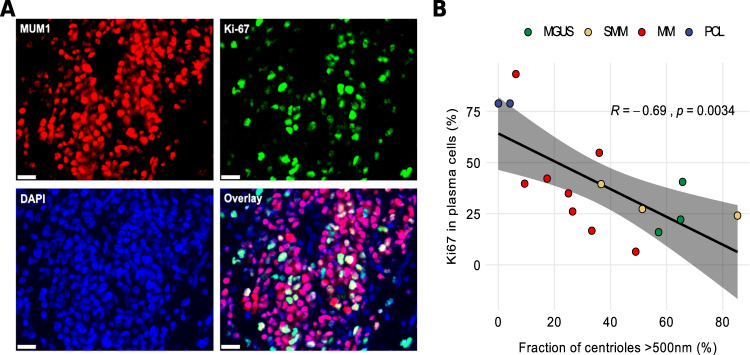
Centriole over-elongation correlates inversely with proliferative activity of CD138^pos^ plasma cells in patients with PCD. **A** Example of correlative immunostaining for MUM1 (red), Ki-67 (green), and DAPI (blue) to assess proliferation activity in a patient with MM. **B** Scatter plot of the fraction of over-elongated centrioles versus plasma cell proliferation rate in 16 patients with plasma cell disorders. The fitted line, *R* and *p* values were obtained by linear regression. N (biological samples): MGUS (3), SMM (3), MM (8), PCL (2).

To establish a correlation between centriole length and proliferative activity, we assessed proliferation rates of plasma cells in 16 out of 21 patients with PCD for whom bone marrow histology samples were available, by co-immunostaining against MUM1 and Ki67 (Fig. 2A). Expectedly, proliferation rates were higher in more advanced disease stages and highest in patients with PCL (Fig. 2B). Correlation with centriole length indicated an inverse relationship between proliferation activity and centriole over-elongation rate (R = – 0.69, *p* = 0.003) (Fig. 2B, Table 2, Supplementary Table S2). In addition, clinical parameters indicating higher plasma cell burden, including increased serum levels of free light chains and bone marrow plasmacytosis, were more pronounced in patients whose cells displayed less centriole over-elongation and higher proliferation rates, thereby suggesting that in cells from PCD patients, centriole over-elongation is associated with less advanced disease (Supplementary Table S4).

### Centriole over-elongation is associated with favorable prognosis in multiple myeloma and plasma cell leukemia

**Fig. 3:**
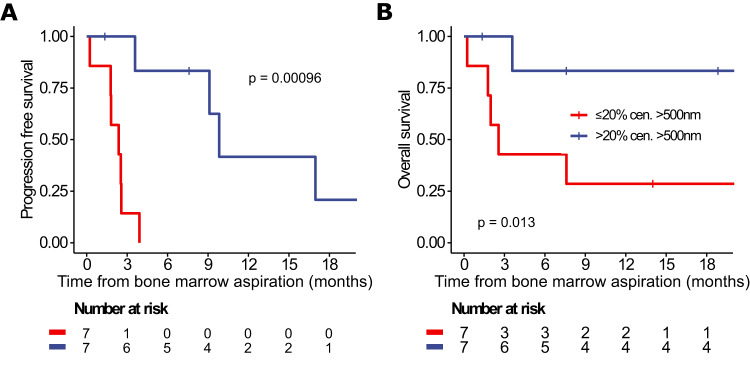
Plasma cell disorder patients with over-elongated centrioles show favorable progression-free and overall survival. **A** Progression-free and **B** overall survival of MM and PCL patients depending on centriole over-elongation. Survival is shown from initial diagnosis of the plasma cell disorder. Comparison between groups was made with the log-rank test. See also Supplementary Fig. S1 and Supplementary Table S4.

The median fraction of over-elongated centrioles was 24% in MM and 4% in PCL patients (Table 2). In this subgroup, patients with >20% over-elongated centrioles showed significantly better progression-free and overall survival as compared to patients with fewer over-elongated centrioles, both for the observational periods starting at initial diagnosis of the PCD and at the time point of bone marrow aspiration for centriole length determination (Fig. 3, Supplementary Fig. S1). Moreover, PCD patients with >20% over-elongated centrioles harbored significantly smaller plasma cell clones, measured as the percentage of plasma cells in the bone marrow (24% vs. 72%, *p* = 0.006). Interphase fluorescence in situ hybridization analysis further revealed a significantly higher frequency of patients with hyperdiploidy in the group with >20% over-elongated centrioles (50% vs. 0%, *p* = 0.044) (Supplementary Table S4).

### Over-elongated centrioles frequently harbor additional structural aberrations

**Fig. 4:**
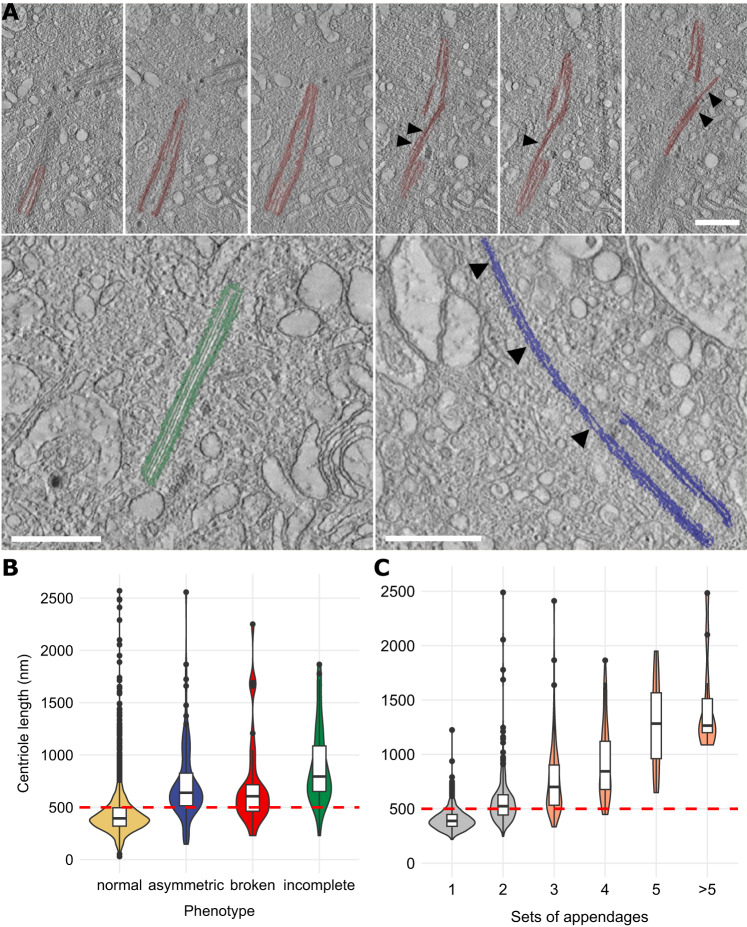
Over-elongated centrioles in human plasma cells show additional structural abnormalities and are frequently fragmented. **A** Exemplary visualizations of broken (top row), incomplete (bottom left), and asymmetric centriole phenotypes (bottom right). Phenotypes were additionally marked with arrow heads. Scale bar, 500 nm. **B** Violin plot and integrated box plots showing the length distribution of normal versus broken, asymmetric, and incomplete centrioles in CD138^pos^ plasma cells from healthy donors and patients with plasma cell disorders. The cut-off value for over-elongation of 500 nm is displayed as red dashed line. **C** Distribution of centriole length depending on the number of appendages. The cut-off value for over-elongation of 500 nm is displayed as red dashed line. Appendages with equal distance from the distal end on the longitudinal axis of a mother centriole were subsumed as “Set of Appendages”.

Recently, we described structural abnormalities including fragmentation of centrioles in a patient with relapsed-refractory multiple myeloma [20]. Exemplary illustrations of the established phenotypes are provided in Fig. 4A. Manual assessment of ET volumes by one experienced observer revealed the same aberrations in healthy donor plasma cells with 1.5% of centrioles being broken (range, 0.0–10.5%), 14.3% being incomplete (range, 6.7–26.3%), and/or 21.2% of centrioles being asymmetrically elongated at their distal ends (range, 8.3–39.5%) (Table 2, Supplementary Tables S2 and S3). The presence of abnormal phenotypes was almost completely restricted to plasma cells with over-elongated centrioles, resulting in a decline of fractions of abnormal centrioles from healthy donor to leukemic plasma cells (Fig. 4B, Table 2). No aberrant phenotypes were detected in CD138^neg^ bone marrow mononuclear cells, U2OS cells, or B-ALL and B-CLL samples (Table 2). In addition, the number of appendages, which were mostly not located at the distal end of the centriole but frequently in the center, increased with the length of centrioles (Fig. 4C, Table 2, Supplementary Table S2).

### Low mRNA expression levels of the centriolar scaffold protein CEP350 are associated with better survival in MM patients

**Fig. 5:**
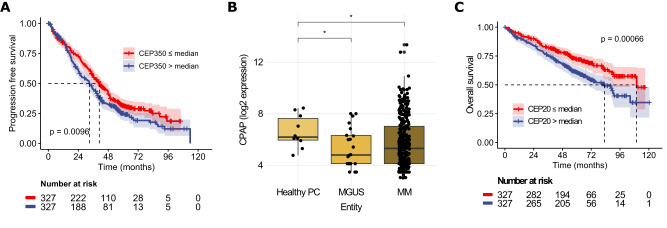
Gene expression analysis of centriolar proteins in PCD patients and their correlation with survival in MM patients. **A** Progression-free survival of 654 MM patients treated in the HD4 and MM5 trials depending on CEP350 mRNA expression levels. Shaded areas indicate 95% CI estimates. **B** mRNA expression levels of the centriole elongation factor CPAP in CD138^pos^ plasma cells from 10 healthy individuals, 22 patients with MGUS and 331 patients with MM. Gene expression represents log2 of GCRMA normalized data using the 223513_at probe. **C** Overall survival of 654 MM patients treated in the HD4 and MM5 trials depending on mRNA expression levels of CEP20, a centriolar elongation inhibitor. Shaded areas indicate 95% CI estimates.

Apart from its essential role in ciliogenesis [12], the centrosomal protein CEP350 was recently shown to function as a central scaffold protein required for centriole wall stability and length control, and coordination of distal centriole end properties. In cell lines, knockout of CEP350 produced over-elongated centrioles with assembly defects at their distal ends, comparable to what we observed in primary CD138^pos^ plasma cells [25]. Gene expression analysis revealed no significant difference in CEP350 levels between plasma cells from healthy controls and patients with MGUS or MM (Supplementary Table S5). In a cohort of 654 MM patients from the GMMG HD4 and MM5 studies [23], however, low CEP350 expression levels were associated with significantly better progression-free (*p* < 0.01) and overall survival (*p* = 0.02) (Fig. 5A, Supplementary Fig. S2).

### mRNA levels of centriole elongation activators are increased in CD138^pos^ plasma cells from healthy donors as compared to MGUS and MM

Centriole length is further controlled by two antagonistic classes of proteins, centriole elongation activators and inhibitors [6, 7]. Expression analysis of the genes belonging to these classes revealed elevated mRNA levels of the centriole elongation activator CPAP and its accessory proteins rotatin, CEP295, and C2CD3 in CD138^pos^ plasma cells from healthy individuals as compared to MGUS and MM (Fig. 5B, Supplementary Fig. S3, Supplementary Table S5). Conversely, although not quite significantly, mRNA levels of the centriole elongation inhibitors CEP20 and CEP97 were elevated in plasma cells from MGUS and MM patients (Supplementary Table S5). Additionally, analysis of expression levels of both genes in a cohort of 654 MM patients treated within the GMMG HD4 and MM5 trials revealed that high expression levels of CEP20 and CEP97 were associated with poorer overall survival (Fig. 5C, Supplementary Fig. S4), reminiscent of our findings for the scaffold protein CEP350.

## Discussion

The incidence of PCD is strictly age-dependent [26]. As only little is known about the early events of PCD evolution, the underlying mechanism for this association has not been determined yet. We here show that over-elongated centrioles accumulate with age in plasma cells from healthy donors. Normal CD138^pos^ plasma cells are terminally differentiated and, in contrast to the majority of other cell types of the bone marrow, survive for several decades in G_1_ phase arrest [27]. In contrast to CD138^pos^ plasma cells, over-elongated centrioles were virtually absent from CD138^neg^ bone marrow mononuclear cells of healthy donors, which mainly constitute fast cycling immune cells and erythroid progenitors, thereby suggesting that centriole over-elongation occurs during prolonged cell cycle arrest. In line, it has recently been shown that cell cycle arrest leads to centriole over-elongation in different cell lines [9]. Chronic stimulation by infectious or self-antigens can initiate the development of MGUS and MM via stimulation of plasma cell proliferation [28]. Primary genomic events involved in myelomagenesis are either hyperdiploidy, defined as the gain of multiple whole chromosomes resulting from errors during cell division, or translocations affecting the immunoglobulin heavy chain locus [17, 18]. Centrosome aberrations are believed to particularly contribute to the development of numerical CIN via induction of aberrant multipolar or clustered, pseudo-bipolar mitoses [2–4]. Accordingly, we found that centriole over-elongation was significantly more frequent in numerically highly aberrant, hyperdiploid PCD samples.

Analogous to over-elongated centrioles, an age-dependent accumulation of somatic chromosomal and mutational abnormalities has been described to occur in various normal tissues including esophagus, skin, colon, bronchus, endometrium, urothelium and blood [29–43]. Clonal hematopoiesis of indeterminate potential (CHIP), defined by a clonal population of blood cells bearing a point mutation in a gene that is recurrently mutated in hematologic malignancies, has been associated with risk of myeloid malignancies [29, 31, 32, 44, 45]. Interestingly and fitting to our observations on over-elongated centrioles as a potential cause of CIN, the development of lymphoid malignancies has been primarily associated with the age-dependent expansion of clonal chromosomal alterations instead of point mutations [30, 33, 46–48]. Similar to healthy plasma cells, the fraction of cells in PCD that actively cycle is very low, particularly in early disease stages. However, in advanced stages and especially in PCL, the fraction of cycling cells considerably increases, thereby explaining the decreased length of centrioles in MM and PCL. Also, and in line with the lack of centriole over-elongation in B-CLL and B-ALL, cell turnover rates in these malignancies are comparatively high with cell survival times in the range of days to months [49]. Accordingly, assessment of bone marrow histologies from 16 PCD patients revealed an inverse relationship between centriole length and plasma cell proliferation rate as determined by co-immunostaining with antibodies to MUM1 and Ki67. Concordantly, plasma cell clone sizes were significantly smaller in PCD patients harboring >20% over-elongated centrioles.

Together, these data suggest that centrioles seem to lengthen with individual cellular age in vivo. The induction of unscheduled cell division of long-lived healthy plasma cells containing over-elongated centrioles may cause chromosome mis-segregation as an initiating event in PCD, and subsequently leads to normalization of centriole length in successive generations of proliferating, malignant plasma cells. Along the same lines, some driver mutations that contribute to clonal expansion in normal tissues seem to undergo negative selection during carcinogenesis in several tissues including esophagus, colon, and blood [35, 50–52]. Further supporting the link between centriole length and proliferation, MM patients with >20% over-elongated centrioles have a significantly better progression-free and overall survival as compared to patients with fewer over-elongated centrioles.

Our expression profiling data reveal elevated mRNA levels of the centriole elongation activator CPAP and its accessory proteins rotatin, CEP295, and C2CD3 in CD138^pos^ plasma cells from healthy individuals as compared to MGUS and MM. CPAP causes slow and progressive elongation of centriolar microtubules [7]. In tissue culture, its overexpression induces abnormally long centrioles, which are fragmented, incomplete, and asymmetric at their elongated distal ends, and contain supernumerary, randomly positioned subdistal appendages [6], highly reminiscent of our findings in plasma cells. Moreover, a non-coding variant upregulating plasma cell expression of CEP120, another CPAP-associated centriole elongation activator and appendage assembly factor [53, 54], has recently been shown to increase MM susceptibility [55].

Conversely, mRNA levels of the centriole elongation inhibitors CEP97 and CEP20 were elevated in PCD plasma cells and associated with poor survival in MM patients [23]. Consistently, a genome-wide association study has shown that single nucleotide polymorphisms, which cause higher expression levels of CEP20, are associated with an increased centrosome index as a surrogate marker of centrosome aberrations and poor survival in MM [14, 56]. For centriole elongation activators, no impact on survival was found, possibly because elongation inhibitors suppress the effects of activators [6, 7]. Together, these data further support our notion of centriole over-elongation in healthy plasma cells and decreasing centriole length in PCD.

In line with these findings, our gene expression profiling data show that low mRNA levels of CEP350 correlate with better progression-free and overall survival in MM patients. CEP350 acts as a central scaffold protein in maturing centrioles and localizes between distal and subdistal appendages [12, 57]. As loss of CEP350 leads to over-elongated centrioles with disturbed stability and architecture [25], our data suggest that upregulation of CEP350 expression with consequential normalization of centriole length might play a role in regaining proliferative potential of malignant plasma cells.

Centrosome aberrations can be subdivided into numerical and structural alterations [5]. Although structural and numerical centrosome aberrations are conceptually distinct, they are similarly prominent and often occur together in tumors. The consequences of numerical centrosome aberrations have been studied extensively. They have been demonstrated to be a major cause of chromosome mis-segregation, implying that these anomalies are likely to contribute to aneuploidy and CIN in human cancers [2–4]. Conceivably, centrosome amplification can promote tumorigenesis in mouse models [58], but evidence for the lack of such an effect also exists [59]. This apparent controversy likely results at least in part from strong selection against extra centrioles in proliferating cells [60].

As compared to the effects of extra centrosomes, structural centrosome aberrations have been less well studied. Nevertheless, recent data suggest a contribution of this aberration type to several aspects of tumorigenesis including CIN as well [6, 8, 9]. A recent screen identified centriole length deregulation as a recurrent structural aberration type in the NCI-60 panel of human cancer cell lines [8]. In vitro, over-elongated centrioles over-accumulated pericentriolar material, perturbing the symmetry of mitotic spindles. Also, they contributed to the generation of supernumerary centrosomes through fragmentation and generation of multiple procentrioles along their elongated walls, leading to subsequent multipolar and clustered, pseudo-bipolar spindle formation and CIN [2, 6, 8]. In addition, increased microtubule anchorage at super-numerary subdistal appendages of over-elongated centrioles might cause chromosome mis-segregation in asymmetric mitotic spindles. Our data presented here show that, in contrast to numerical centrosome aberrations, centriole over-elongation and subsequent structural centrosome aberrations can develop in non-proliferating, quiescent cells, thereby circumventing negative selection, and suggest that negative selection resumes once plasma cell proliferation is initiated. As re-induction of mitosis in quiescent, post-mitotic primary human plasma cells is experimentally not established, this hypothesis can unfortunately currently not be tested.

Using a semi-automated high-throughput workflow with cutting-edge ET, we have analyzed the centrosomal phenotype of primary healthy, premalignant, and malignant bone marrow plasma cells at the ultrastructural level on a high scale. In accordance with the recent discovery that centrioles do not have an elongation monitoring mechanism [9], we have found that in long-lived, post-mitotic plasma cells over-elongated centrioles accumulate in an age-dependent manner. As the percentage of over-elongated centrioles successively decreased in increasingly aggressive stages of PCD, resumption of cell division might first contribute to the generation of chromosome aberrations, which are characteristic of early PCD stages already, and subsequently lead to normalization of centriole length in successive generations of proliferating, malignant plasma cells. Structural centrosome aberrations may thus contribute to initial oncogenesis rather than to disease progression in PCD. This might similarly hold true for malignancies originating from rarely dividing stem cells.

### Supplementary information

Supplemental Material

Supplemental Table 1

Supplemental Table 2

Supplemental Table 3

Supplemental Table 4

Supplemental Table 5

## Data Availability

A more detailed method description is provided in the supplemental materials, available on the *Leukemia* website. ET data have been deposited to EMPIAR (IDs: 11243 and 11505, respectively). De-identified patient genome expression data can be accessed from ArrayExpress (Accession No.: E-MTAB-81/E-GEOD-2658). For requests regarding additional data, please contact a.kraemer@dfkz-heidelberg.de.
