# Supplementary material for: High-throughput electron tomography identifies centriole over-elongation as an early event in plasma cell disorders
Source: Leukemia. 2023 Oct 11;37(12):2468–78. doi: 10.1038/s41375-023-02056-y (PMC10681902; doi:10.1038/s41375-023-02056-y)
Supplement: Supplementary file 1 — Supplemental Material [file 41375_2023_2056_MOESM1_ESM.pdf]

Supplemental Materials to ‚High-throughput electron tomography identifies centriole over-elongation as an early event in plasma cell disorders‘

Sebastian Köhrer,[1,2,**] Tobias Dittrich,[1-4,**] Martin Schorb,[5] Niels Weinhold,[3] Isabella Haberbosch,[1,3] Mandy Börmel,[5] Gabor Pajor,[1,6] Hartmut Goldschmidt,[3,7] Carsten Müller-Tidow,[3,8] Marc S. Raab,[1,3] Lukas John,[1,3] Anja Seckinger,[3] Alexander Brobeil,[9] Peter Dreger,[3] Tamás Tornóczky,[6] László Pajor,[6] Ute Hegenbart,[3.4] Stefan O. Schönland,[3,4] Yannick Schwab,[2,5,*] and Alwin Krämer[1,3,*]

[1]Clinical Cooperation Unit Molecular Hematology/Oncology, German Cancer Research Center (DKFZ) and Department of Internal Medicine V, University of Heidelberg, Heidelberg, Germany; [2]Cell Biology and Biophysics Unit, European Molecular Biology Laboratory (EMBL), Heidelberg, Germany; [3]Department of Internal Medicine V, University of Heidelberg, Heidelberg, Germany; [4]Amyloidosis Center, University of Heidelberg, Heidelberg, Germany; [5]Electron Microscopy Core Facility, European Molecular Biology Laboratory (EMBL), Heidelberg, Germany; [6]Department of Pathology, University of Pécs Medical School and Clinic, Pécs, Hungary; [7]Department of Internal Medicine V, GMMG-Studygroup at University of Heidelberg, Heidelberg, Germany; [8]National Center for Tumor Diseases (NCT), University of Heidelberg, Heidelberg, Germany; and [9]Institute of Pathology, University of Heidelberg, Heidelberg, Germany

*Y.S. and A.K. jointly supervised this study.
** These authors contributed equally.

SUPPLEMENTAL METHODS

Plasma cell disorder patients and healthy donors

All patients and healthy donors gave written informed consent. The study was performed in accordance with the principles of the Declaration of Helsinki. The Ethics Committee of the University of Heidelberg, the European Molecular Biology Laboratory (EMBL) review board, and the Research Ethics Committee of the University of Pécs all approved the study (Ethics Committee of the University of Heidelberg approval reference number: S-206/2011; EMBL BIAC application number: 2019-005; Ethics Committee of the University of Pécs reference number: BMEÜ/4146/2022/EKU).

Cell lines

U2OS cells (ATCC HTB-96) were obtained from ATCC (Manassas, Virginia, USA) and were kept in a humidified incubator at 37 °C and 5% CO_2. Overexpression of polo-like kinase 4 in U2OS cells was achieved as described previously(1). U2OS cells (both control and PLK4-induced) were cultured in DMEM Glutamax medium (31966047, Life Technologies, Carlsbad, California, USA) supplemented with 10 % fetal bovine serum (FBS) (631106, Clontech, Mountain View, California, USA), 100 µg/ml hygromycin B (10687010, Life Technologies), and 1.5 µg/ml puromycin (2600023, Life Technologies). Cells were passaged 1:10 at 80 % confluency.

Sample acquisition and isolation of target cells in PCD patients and healthy donors

Bone marrow aspiration was performed according to clinical standard operating procedures at the Department of Internal Medicine V, University of Heidelberg. Five additional bone marrow samples from patients with B-ALL were obtained according to clinical standard operating procedures at the University of Pécs Medical Center. Ficoll gradient centrifugation was used to isolate bone marrow mononuclear cells. Subsequently, $CD138^{pos}$ plasma cells were separated by magnetic-activated cell sorting (MACS). Fluorescence-activated cell sorting (FACS) revealed a mean plasma cell purity of 88.9 ± 9.6 %. $CD138^{neg}$ bone marrow mononuclear cells of healthy controls were preserved and prepared for both immunofluorescence imaging and electron tomography. In patients with PCL, plasma cells were directly isolated from peripheral blood. In one case with PCL, plasma cells were additionally isolated from a bone marrow aspirate, and this was treated as additional biological sample. If the sample size was larger than 300,000 cells, samples were asymmetrically split and prepared for both immunofluorescence and electron tomography.

Sample acquisition and isolation of target cells in patients with B-ALL and B-CLL

For 1 B-ALL and all B-CLL samples, peripheral blood was drawn from patients with either B-ALL or B-CLL. Cells were washed with PBS. After centrifugation, lymphocytes were isolated using a specific separation medium (GTF1511KYA, Linaris, Dossenheim, Germany). After another washing step, red cell lysis of the remaining erythrocytes was achieved by centrifugation, removing supernatant, and adding 5 ml of red cell lysis buffer (155 mM NH4Cl, 10 mM KHCO3 and 0.1 mM EDTA in de-mineralized purified water) and incubating cells in the dark for 10 minutes at room temperature. Afterwards, cells were washed and resuspended in PBS.

Induction of centrosome amplification in U2OS fibroblasts stably overexpressing Polo-like kinase 4 (PLK4)

For induction of centrosome amplification, U2OS-PLK4 cells were trypsinized, resuspended in culture medium with a final concentration of 12 x 10^4 cells per ml and supplied with 2 µg/ml tetracycline (T7660-5G, SigmaAldrich, St. Louis, Missouri, USA). Cells were induced for 16 hrs (one replicate) and 40 hrs (two replicates), respectively.

Light microscopy and immunofluorescence imaging

$CD138^{pos}$ plasma cell samples from patients with plasma cell disorders and healthy control donors, $CD138^{neg}$ bone marrow mononuclear cells from three healthy control donors, B-ALL and B-CLL patient samples, and induced as well as non-induced U2OS-PLK4 cells were prepared for immunofluorescence microscopy analysis of numerical centrosome aberrations. Cells were washed in PBS and approximately 100,000 cells were spun onto slides. For fixation, cells were incubated in 100 % IF-grade methanol at -20 °C for 10 minutes. After incubation with blocking buffer (10 % goat serum in PBS) for 20 minutes, cells were incubated with primary antibodies against centrin (mouse; 04-1624, Merck Millipore, Burlington, Massachusetts, USA) and pericentrin (rabbit; ab4448, Abcam, Cambridge, UK) in blocking buffer for one hour. Afterwards, samples were washed with PBS three times, followed by incubation with secondary antibodies (Alexa Fluor 488 (A11029, Invitrogen, Waltham, Massachusetts, USA) and 568 (A11036, Invitrogen), respectively) for 30 minutes. Thereafter, cells were washed again with PBS and nuclei staining was achieved with Hoechst (H3570, Invitrogen). After three more washing steps with PBS, bidistilled water and 100 % ethanol, samples were mounted onto coverslides with Vectashield mounting medium (H-1000, Vector Laboratories, Newark, California, USA). All incubation steps were performed at room temperature.

Immunofluorescence imaging was done on the same or the following day on a Zeiss Cell Observer with a 40x 1.3 Plan Apochromat objective using ZEN blue 2.6 (Carl Zeiss Microscopy, Oberkochen, Germany). For evaluation of numerical centrosome aberrations, at least 100 cells per sample were classified into two groups: Cells with four or less signal maxima in the centrin channel, and two or less signal maxima in the pericentrin channel were deemed normal. If the number of maxima was higher in one or two of the two channels, cells were deemed amplified. Cells were excluded from the analysis if they were out of focus, showed no distinctive signal in one of the two channels, or overlapped with other cells.

For evaluation of primary cilia formation, BJ fibroblasts and cells from one healthy control donor were used. To induce primary cilia formation in BJ fibroblasts, cells were grown on coverslips and starved in 0.1 % FBS-containing medium for 48 hours prior to fixation. Starved and non-starved BJ fibroblasts served as positive and negative controls, respectively. After washing with PBS, cells were fixed with 4% paraformaldehyde for 15 minutes at room temperature. Permeabilization of cell membranes was achieved by adding 0.2 % Triton-X-100 (HFH10, ThermoFisher, Waltham, Massachusetts, USA) in PBS to the coverslips and incubating for five minutes at room temperature, followed by washing twice in PBS. Cells were blocked for 20 minutes and then stained with primary antibodies against ARL13B (rabbit; 17711-1-AP, ProteinTech, Manchester, UK) and poly-glutamylated tubulin (mouse; AG-20B-0020B, AdipoGen, San Diego, California, USA). All subsequent steps were equal to numerical centrosome aberration evaluation as described above.

Immunofluorescence and immunohistochemical data generated during this study will be shared by the lead contact upon request.

Electron tomography acquisition, data reconstruction, and analysis

A detailed description of our electron microscopy workflow was published recently.(2, 3) In brief, CD138^{pos} plasma cells from patients with plasma cell disorders and healthy control donors, CD138^{neg} bone marrow mononuclear cells from three healthy control donors, and U2OS cells were fixed with EM fixative consisting of 2.5 % EM-grade glutaraldehyde (16220, Electron Microscopy Sciences, Hatfield, Pennsylvania, USA) and 2 % paraformaldehyde (15710, Electron Microscopy Sciences) in 0.1 M cacodylate buffer over night. On the next day, fixative was removed, cells were stained with 1 mg/ml Evans Blue in 0.1 M cacodylate buffer and pre-embedded into 2 % low melting point agarose (161-3111, Bio-Rad, Hercules, California, USA) to form a visible cell pellet. Post-fixation and contrast enhancement was achieved with 1 % osmium tetroxide (19150, Electron Microscopy Sciences) in bidistilled water (dH_2O) and 1 % uranyl acetate in dH_2O (77870, Serva, Heidelberg, Germany), followed by

dehydration with an increasing acetone series. After infiltration with Epon epoxy resin 812 (hard formula; 21045, 20755, 29452, and 36975, Serva), cell pellets in resin were transferred to an embedding mold and polymerized at 60 °C for 48 hours. Post-fixation, dehydration, and Epon infiltration were performed using a temperature-controlled microwave (BioWavePro, Pelco, Fresno, California, USA) equipped with a vacuum pump at 23 °C. The resulting blocks were trimmed and serially sectioned with a UC7 ultramicrotome (Leica Biosystems, Wetzlar, Germany) equipped with a diamond knife (Diatome). Five or more consecutive sections of 200 nm thickness each were collected and loaded onto formvar-coated slot grids. Sample preparation was concluded with post-staining of several grids using 2 % uranyl acetate and lead citrate. Electron microscopy experiments were performed on a JEOL JEM 2100Plus equipped with a JEOL Matataki sCMOS camera (JEOL, Akishima, Japan) and a Tecnai F30 (ThermoFisher) equipped with a Gatan OneView camera (Gatan, Pleasanton, California, USA). Both electron microscopes were operated with SerialEM(4) software. We used a high-throughput transmission electron microscopy workflow to screen for target cells containing centrosomes(2, 3, 5). Firstly, we obtained a montaged image of the central section of the grid at 400x magnification. Secondly, extrapolation of the image data with KNIME (Konstanz Information Miner)(6) and pyEM was used to label individual cells on the section. Lastly, we automatically acquired images of each labelled cell at 1000x and 3000x magnification, respectively, utilizing a custom script and SerialEM's advanced navigator functionality. Manual evaluation of 3000x magnification images was performed by one experienced user to identify cells containing centrosomal structures. These cells were then semi-automatically targeted on the remaining sections of the grid. After labelling of exact positions of centrosome-containing regions in SerialEM, single axis electron tomography was performed automatically at 15,500x magnification (1.55 nm/px, tilt range -60° to +60°, increment 1°).

Raw tomography data was reconstructed on a high-performance computer cluster using the batch tomogram reconstruction functionality of the IMOD software package(7), and matching tomograms of one cell on different sections were joined manually using etomo(8). Thereby, 3D volumes of approximately 3.1 x 3.1 µm in X and Y, and at least 1 µm in Z dimension were created. Electron tomography data generated during this study have been deposited at EMPIAR (EMPIAR-ID: 00000), and epoxy resin blocks for electron microscopy experiments of all patients and cell lines analyzed in this study are available upon request from the lead contact.

For further analysis, only centrioles that were completely displayed within the created volume were considered. Measuring and annotation of electron tomography data was achieved using IMOD's model feature and was entirely done by S.K. alone to ensure comparability. Measuring

of centrioles comprised its longitudinal axis and its diameters at the proximal and distal end and at the centre. In mother centrioles, all appendages and subdistal appendages were labelled. If a centriole, on the other hand, did not harbour appendages, it was deemed ‘daughter’.
Additionally, a fraction of centrioles showed structurally aberrant phenotypes as described by us recently(2). We labelled these phenotypes

i. asymmetric, if the typical 9x3 architecture of an ordinarily configurated centriole was only displayed on one of the centriole’s ends, but it consisted of less than nine triplets on the other, and/or
ii. incomplete, if the typical 9x3 cylinder shape was never found along the centriole's longitudinal axis, but the structures clearly resembled centriole features (e.g., less than nine microtubule triplets carrying appendages), and/or
iii. broken, if the typical 9x3 configuration was preserved, but showed diverging triplets on one end or other clear breakage points.

Data extraction from IMOD-files and statistical analysis

After measuring and annotation, centriole coordinates, lengths, diameters as well as appendage coordinates and, if applicable, phenotype annotations were extracted from model files and the output was generated as a table using a custom KNIME workflow. Tables were generated for individual patients and respective subgroups. These tables were used for statistical analysis which was performed in R statistical environment 3.5.3[(9)] on a x86_64_w64_mingw32/x64 (64-bit) platform. Additionally, the R ‘survival’ package[(10, 11)] was used. For all experiments, results with p-values not larger than 5% were considered statistically significant.

Non-normal distribution of centriole lengths was confirmed in each subgroup with Shapiro-Wilk test. Thus, only non-parametric statistics were applied for analysis of centriole characteristics. Continuous data were described with median and range. Median length or diameter of biological replicates were used to calculate median and range of centriole length or diameter and to compare parameters between groups. To display the distribution of centriole measures within subgroups (Figure 1A-D, Figure 4A-B), individual centriole measures were used irrespective of biological replicates. To test differences in continuous variables, the Wilcoxon rank sum test was used for pairwise comparisons and the Kruskal-Wallis rank sum test was used for multiple comparisons, whereas Fisher’s exact test was used to test differences in categorical variables between groups. Kaplan-Meier estimates were used to describe overall survival and the log rank test was applied for comparisons. All statistical tests were two-sided.

As we observed no significant change in the centriole structure or phenotype between U2OS-PLK4 induced for 16 hours and 40 hours, the respective biological samples were subsumed under the cell type “U2OS-PLK4” and not further distinguished.
One selected tomogram was manually segmented in Amira-Avizo software platform version 2022.1 (ThermoFisher), using the threshold-based brush segmentation tool (Figure 1E).

Multimodal big image sharing and exploration (MoBIE)
Electron tomograms generated during this study can be interactively visualized in ImageJ(12) using the recently published plugin MoBIE(13). This plugin provides a cloud-based image data hosting tool to display and interactively browse large (terabyte-scale) datasets requiring only a standard PC with stable internet access. Reconstructed tomography data was converted into BDV-N5 format and model coordinates of longitudinal centriole axes were used to produce different display options of the data. For general instructions on how to set up and use MoBIE on your PC, refer to the following link: https://github.com/mobie/mobie-viewer-fiji. To display data described in this paper, open MoBIE in ImageJ and open the project ‘https://github.com/mobie/centrioles-tomo-datasets’. Using the ‘Tomograms’ dropdown menu, reconstructed volumes can be visualized labelled according to entity and patient number, while individual centrioles can be displayed along their longitudinal axis via selection from the ‘Centrioles’ dropdown menu.

Gene Expression Profiling
RNA extraction was performed using the RNeasy Mini Kit (74104, Qiagen, Venlo, Netherlands), the SV Total RNA Isolation System (Z3101, Promega, Madison, Wisconsin, USA) and Trizol RNA extraction reagent (15596026, Invitrogen). Labeled cRNA was generated using the small sample labeling protocol vII (Affymetrix, Santa Clara, California, USA), and hybridized to GeneChip™ Human Genome U133 Plus 2.0 Arrays (900466, Applied Biosystems, Waltham, Massachusetts, USA), according to the manufacturer’s instructions. For comparison of expression values in normal, pre-malignant and malignant bone marrow plasma cells we used the hgu133plus2cdf as chip definition file (CDF). For expression data of clinical study patients from the HD4 and MM5 trials(14), we used the Affymetrix U133 Version 2.0 plus array custom CDF (v25) mapping to Entrez genes (http://brainarray.mhri.med.umich.edu/Brainarray/Database/CustomCDF/). Expression data were normalized using GC-RMA. Absolute gene expression levels are displayed as log_2-transformed values. De-identified patient genome expression data have been deposited at ArrayExpress and are publicly available as of the date of publication under the accession numbers E-MTAB-81 / E-GEOD-2658.

Semi-automated MUM1-based quantification of histological sections

For immunohistochemical analyses, a representative tissue block containing MUM1-positive MM cells was selected. The blocks were sectioned with a standard microtome at 2 μm thickness. Subsequently, the slides were dried overnight at room temperature. Immunohistochemical staining for MUM1 (rabbit anti-human, clone MRQ-43) (358R-74, Cellmarque, Rocklin, California, USA) and Ki-67 (mouse anti-human, clone MIB-1) (GA626, Agilent DAKO, Santa Clara, California, USA) was performed using the automated immunostainer LINK48 (Agilent DAKO) with the corresponding secondary antibodies: Alexa Fluor 488 for MUM1 visualization and Alexa Fluor 568 for Ki-67 visualization (A11017 and A11011, respectively, Invitrogen). Subsequently, the slides were coverslipped with VectaShield containing DAPI (H-1200, Vector Laboratories). The stained slides of the selected MM cases were digitalized using a Hamamatsu NanoZoomer slide scanner equipped with the appropriate filter sets for detecting Alexa Fluor 488 (FITC channel), Alexa Fluor 568 (TxRed channel) and DAPI (Hamamatsu, Herrsching, Germany). For image acquisition the manufacturer’s acquisition software suite NDP.view2 was used (Hamamatsu).

Bone marrow plasmacytosis was evaluated by manual counting of HE-stained smears according to clinical standards (available for all 21 patients with plasma cell disorders and one healthy donor) as well as by semi-automated MUM1-based quantification of histological sections from bone marrow trephine biopsies (available for 15 of 21 patients with plasma cell disorders). If both cytology and histology were available, the histological results were preferred.

Quantification of MUM1-positive myeloma cells and co-labelling with Ki-67 to determine their proliferation index was done using QuPath 0.2.3(15). The images were imported using the Bioformats builder. The three fluorescent channels were named as FITC (Alexa Fluor 488, MUM1), TXRed (Alexa Fluor 568, Ki-67) and DAPI. Detection of all cells within the specimen was done using the integrated “cell detection” module of QuPath with DAPI as the reference channel. The module additionally estimates the full extent of each cell based upon a constrained expansion of the nucleus region and calculates up to 66 measurements of intensity and morphology. These data were used to create a “single measurement classifier” for detecting MUM1-positive myeloma cells. For classification the following settings were used: object filter: “cells”, channel filter: “TxRed”, measurement: “nucleus:TxRed mean”. The threshold for detection was calculated for each image by analyzing at least 30 myeloma cells for their MUM1 fluorescence signal and the mean value was then formed. Cells above the threshold were classified as “myeloma cells” and cells below the threshold were classified as “hematopoiesis”. The correct classification of myeloma cells was checked by expert pathologists. After detecting the MUM1-positive myeloma cells, their Ki-67 index was analyzed

SUPPLEMENTAL FIGURES

Supplemental Figure S1. Plasma cell disorder patients with over-elongated centrioles show favorable progression-free and overall survival. (A) Progression-free and (B) overall survival of MM and PCL patients depending on centriole over-elongation. Survival is shown from time of bone marrow aspiration for this analysis. Comparison between groups was made with the log-rank test. See also Figure 3 and Supplemental Table S4.

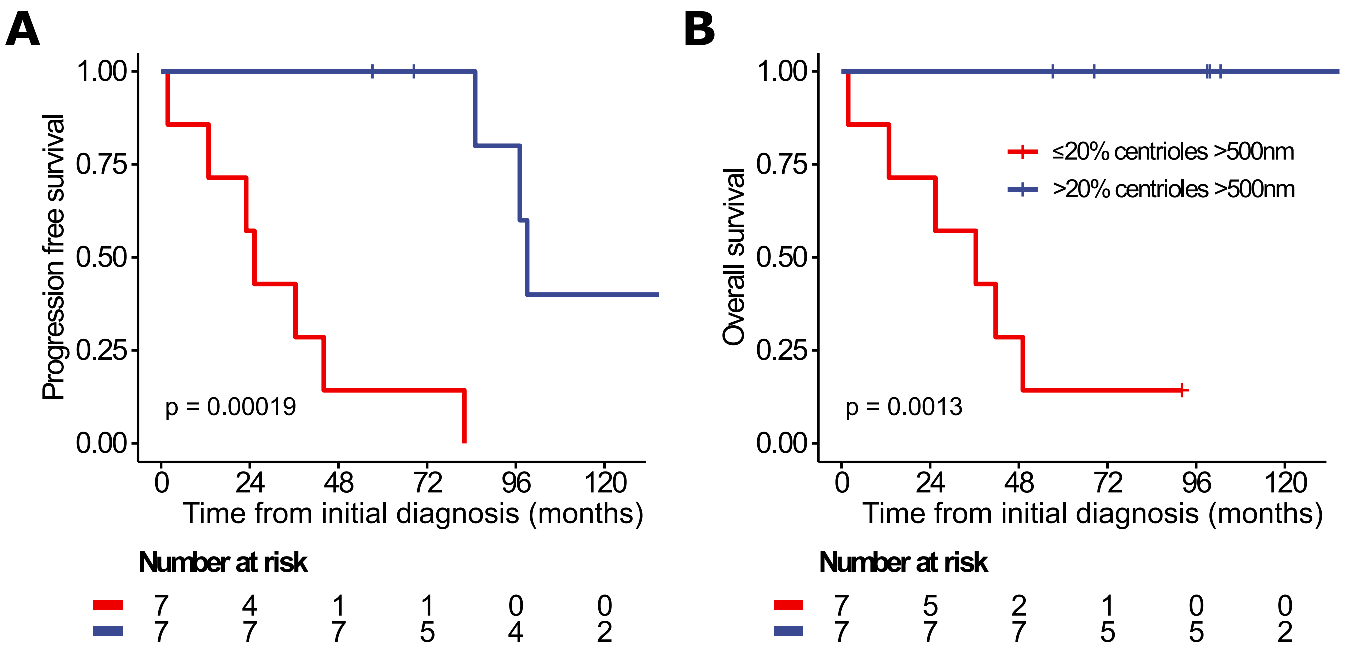

Supplemental Figure S2. CEP350 expression levels of the centriolar scaffold protein CEP350 correlate inversely with survival in MM patients. Overall survival of 654 MM patients treated in the HD4 and MM5 trials depending on CEP350 mRNA expression levels. Shaded areas indicate 95% CI estimates.

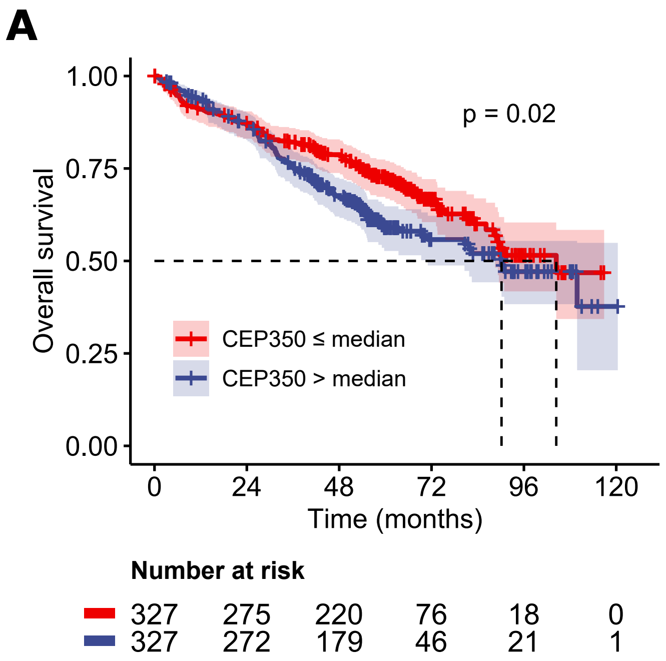

Supplemental Figure S3. Accessory proteins of the centriole elongation factor CPAP show higher expression levels in CD138pos plasma cells from healthy donors as compared to cells from MGUS or MM patients. mRNA expression levels of rotatin (A), CEP295 (B), and C2CD3 (C) were analysed in CD138^{pos} plasma cells from 10 healthy individuals, 22 patients with MGUS and 331 patients with MM, respectively. Gene expression represents log2 of GCRMA normalized data using the following probes: *CPAP,* 223513_at; *rotatin*, 227072_at; *CEP295,* 229878_at; and *C2CD3,* 213199_at. Shaded areas indicate 95% CI estimates.

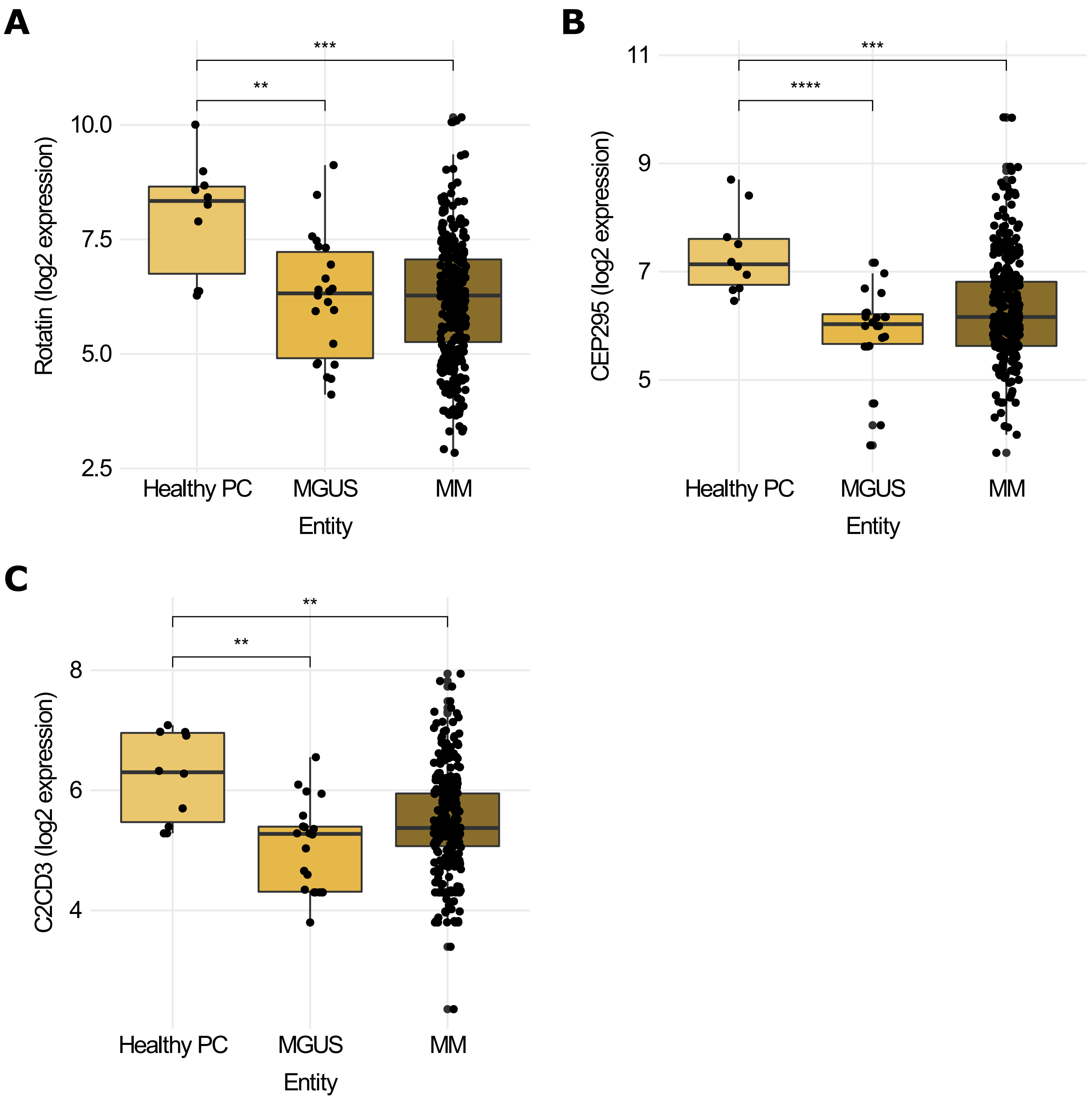

Supplemental Figure S4. High expression levels of CEP97, a centriole elongation inhibitor, correlate with poorer overall survival in MM patients. Overall survival of 654 MM patients treated in the HD4 and MM5 trials depending on CEP97 mRNA expression levels. Shaded areas indicate 95% CI estimates.

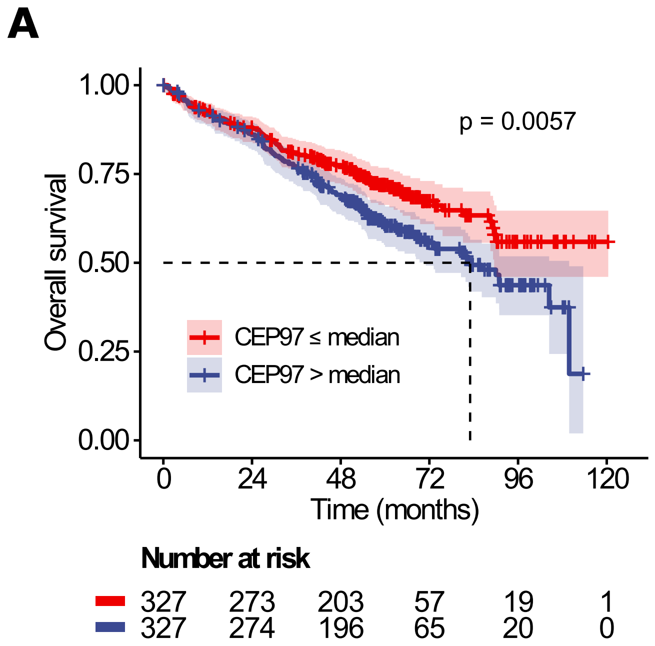

SUPPLEMENTAL TABLES (provided as separate .xlsx-file)

Table S1. Related to Table 1. Clinical characteristics of individual patients and healthy donors.
HDM+ASCT, high-dose chemotherapy with melphalan followed by autologous stem cell transplantation; BM plasmocytosis, fraction of plasma cells in bone marrow; MC, monoclonal component; HC, heavy chain measured in serum; LC, light chain; FLC, free light chains measured in serum.

Table S2. Related to Table 2 and Figure 2. Centriole parameters of individual biological samples.
Centriole lengths and diameters are shown as median [range]. The individual centriole diameter of one centriole is calculated by averaging its proximal, central, and distal diameter. N appendages, the median number of appendages at mother centrioles. *PCL_00 are mononuclear peripheral blood cells and PCL_01 are CD138pos bone marrow cells from the same patient.

Table S3. Related to Table 2. Centriole parameters of patients with plasma cell disorders and controls stratified by mother and daughter centrioles. Data is shown as median [range] of the investigated biological samples. In PCL, for one patient, cells from peripheral blood and bone marrow were analyzed separately. Parameters (e.g., centriole length or maximal centriole length) were first calculated for each individual biological samples. Medians and ranges were then calculated in the respective entities based on these parameters. In case of n=2, the two values given in square brackets represent the individual calculated medians of the parameters. Centrioles abnormal, presence of any of the phenotypes (asymmetric, broken, or incomplete).

Table S4. Related to Table 1 and Figure 4. Clinical characteristics and cytogenetic status of PCD patients stratified by over-elongation of centrioles.
Data are shown as count (% of respective total). No Ki67 data are available for five patients, two of whom with >20% over-elongated centrioles. No FISH data are available for one patient with MGUS and one patient with SM. Chromosomal translocation, t(11;14) or t(4;14) or t(14;16) or t(6;14); Odd gain, any gain of the chromosomes 5p15, 9q34, 15q22, 17p13, or 19q13; Hyperdiploid, two or more gains of chromosomes 5p15, 9q34; or 15q22; High risk, del17p13 or t(4;14) or t(14;16).

Table S5. Related to Figure 5. Gene expression levels of centrosome-related genes in CD138pos plasma cells from healthy donors and patients with PCD.

Expression data were normalized using GC-RMA. p values for comparison between groups were determined using hgu133plus2cdf as chip definition file.

SUPPLEMENTAL REFERENCES

1. Konotop G, Bausch E, Nagai T, Turchinovich A, Becker N, Benner A, et al. Pharmacological Inhibition of Centrosome Clustering by Slingshot-Mediated Cofilin Activation and Actin Cortex Destabilization. Cancer Res. 2016;76(22):6690-700.
2. Dittrich T, Kohrer S, Schorb M, Haberbosch I, Bormel M, Goldschmidt H, et al. A high-throughput electron tomography workflow reveals over-elongated centrioles in relapsed/refractory multiple myeloma. Cell Rep Methods. 2022;2(11):100322.
3. Kohrer S, Dittrich T, Schorb M, Haberbosch I, Schwab Y, Kramer A. Protocol for high-throughput electron tomography exemplified on centrioles in primary human plasma cells. STAR Protoc. 2023;4(3):102373.
4. Mastronarde DN. Automated electron microscope tomography using robust prediction of specimen movements. J Struct Biol. 2005;152(1):36-51.
5. Schorb M, Haberbosch I, Hagen WJH, Schwab Y, Mastronarde DN. Software tools for automated transmission electron microscopy. Nat Methods. 2019;16(6):471-7.
6. Berthold MR, Cebron N, Dill F, Gabriel TR, Kötter T, Meinl T, et al., editors. KNIME: The Konstanz Information Miner. Data Analysis, Machine Learning and Applications; 2008 2008//; Berlin, Heidelberg: Springer Berlin Heidelberg.
7. Kremer JR, Mastronarde DN, McIntosh JR. Computer visualization of three-dimensional image data using IMOD. J Struct Biol. 1996;116(1):71-6.
8. Mastronarde DN, Held SR. Automated tilt series alignment and tomographic reconstruction in IMOD. J Struct Biol. 2017;197(2):102-13.
9. Team RDC. A language and environment for statistical computing. http://www R-project org. 2009.
10. Therneau TM, Grambsch PM, Therneau TM, Grambsch PM. The cox model: Springer; 2000.
11. Therneau TM, Lumley T. Survival: survival analysis. R package version. 2008:2.34-1.
12. Schneider CA, Rasband WS, Eliceiri KW. NIH Image to ImageJ: 25 years of image analysis. Nat Methods. 2012;9(7):671-5.
13. Pape C, Meechan K, Moreva E, Schorb M, Chiaruttini N, Zinchenko V, et al. MoBIE: a Fiji plugin for sharing and exploration of multi-modal cloud-hosted big image data. Nat Methods. 2023.
14. Weinhold N, Salwender HJ, Cairns DA, Raab MS, Waldron G, Blau IW, et al. Chromosome 1q21 abnormalities refine outcome prediction in patients with multiple myeloma - a meta-analysis of 2,596 trial patients. Haematologica. 2021;106(10):2754-8.
15. Bankhead P, Loughrey MB, Fernandez JA, Dombrowski Y, McArt DG, Dunne PD, et al. QuPath: Open source software for digital pathology image analysis. Sci Rep. 2017;7(1):16878.
